# Fc receptor-like 5 gene polymorphisms and mRNA expression are associated with liver fibrosis in chronic hepatitis B

**DOI:** 10.3389/fmicb.2022.988464

**Published:** 2022-09-09

**Authors:** Jiajia Yang, Juan Gu, Hongmei Wang, Jiayin Shi, Lingyun Lu, Wanxian She, Ying Wang

**Affiliations:** ^1^Department of Infection Management, The Affiliated Suzhou Hospital of Nanjing Medical University, Suzhou Municipal Hospital, Gusu School, Nanjing Medical University, Suzhou, China; ^2^Department of Clinical Laboratory, The Affiliated Yancheng Hospital, School of Medicine, Southeast University, Yancheng, China; ^3^Department of Outpatient, The Affiliated Suzhou Hospital of Nanjing Medical University, Suzhou Municipal Hospital, Gusu School, Nanjing Medical University, Suzhou, China; ^4^Department of Gastroenterology, The Affiliated Suzhou Hospital of Nanjing Medical University, Suzhou Municipal Hospital, Gusu School, Nanjing Medical University, Suzhou, China

**Keywords:** Fc receptor-like 5, gene polymorphisms, liver fibrosis, chronic hepatitis B, association study

## Abstract

**Objective**: To investigate the associations of Fc receptor-like 5 (*FCRL5*) gene polymorphisms and mRNA expression with liver fibrosis in chronic hepatitis B (CHB).

**Methods**: A total of 114 CHB patients with liver fibrosis and 120 CHB patients without liver fibrosis were selected for this study. The gender, age, body mass index (BMI), alanine transaminase (ALT) value, aspartate aminotransferase (AST) value, aspartate aminotransferase-to-platelet ratio index (APRI), and fibrosis index based on 4 factors (FIB-4) were recorded. Two polymorphisms of the *FCRL5* gene (rs6427384 and rs6692977) were genotyped. The mRNA expression level of *FCRL5* in peripheral blood monocytes was determined.

**Results**: ALT, AST, APRI, and FIB-4 in patients with fibrosis were significantly higher than those in non-fibrosis patients. There was statistically significant difference between fibrosis and non-fibrosis groups in the genotype distribution (χ^2^ = 7.805, *p* = 0.020) and allele frequencies (χ^2^ = 13.252, *p* < 0.001) at *FCRL5* rs6692977. When compared with CC genotype, the genotype CT or TT at rs6692977 was significantly associated with a increased risk of liver fibrosis in CHB patients (CT vs. CC: OR = 1.921, 95% CI = 1.093–3.375, *p* = 0.023; TT vs. CC: OR = 2.598, 95% CI = 1.067–6.324, *p* = 0.031). The mRNA relative expression levels of *FCRL5* in patients with liver fibrosis were significantly higher than those in the non-fibrosis group (*t* = 13.456, *p* < 0.001). The fibrosis patients carried TT or CT genotype of rs6692977 had significantly higher *FCRL5* mRNA expression levels than those carried CC genotype (*t* = 2.859, *p* = 0.005). The mRNA expression levels of *FCRL5*, APRI, and FIB-4 index showed predictive efficacy in liver fibrosis with cut-off values of 0.75 (AUC = 0.896, 95% CI = 0.856–0.935), 0.45 (AUC = 0.852, 95% CI = 0.802–0.902) and 1.84 (AUC = 0.765, 95% CI = 0.703–0.826), respectively.

**Conclusion**: *FCRL5* gene rs6692977 polymorphisms and mRNA expression levels are associated with liver fibrosis in CHB patients.

## Introduction

Hepatitis B virus (HBV) infection and chronic hepatitis B (CHB) caused by HBV are global public health problems (Jieanu et al., 2015). China has one of the highest HBV prevalence worldwide (Su et al., 2022). Liver fibrosis is the result of persistent inflammatory response and chronic scar healing response in the process of chronic liver injury. Hepatitis B-related fibrosis is an important progression stage of CHB (Zhao et al., 2022). If not timely and effective treatment, liver fibrosis may eventually develop into liver cirrhosis or even hepatocellular carcinoma (HCC), which seriously affects the health and quality of life of patients (Li et al., 2022). Therefore, screening populations and identifying individuals at a high risk of disease progression may be beneficial in translating potential therapeutic targets into clinical practice, thereby improving outcomes (Wu et al., 2015, 2017).

At present, the causes of liver fibrosis in patients with CHB are not yet fully understood. Genetic predisposition, innate immune aspects, and inflammatory response combined with environmental factors were recognized to exert a crucial role in the development of hepatitis B-related liver fibrosis (Yang et al., 2018; Campos et al., 2020; Ma et al., 2021; Naghib and Kariminik, 2022). As one of the most common heritable variations, single nucleotide polymorphisms (SNPs) have attracted the attention of many researchers in recent years. Up to now, many studies have shown abundant gene susceptibility loci associated with HBV infection and related diseases, including interleukin-10 (Guo et al., 2015), Sodium taurocholate co-transporting polypeptide (Su et al., 2022), and RNA-binding protein lin28 gene polymorphisms (Han et al., 2020). Nevertheless, the associations having been reported cannot completely explain the mechanism of hepatitis B-related fibrosis. Thus it is worthwhile to continue to explore the genetic variants and molecular markers associated with this disease.

Fc receptor-like molecules (FCRLs) are a class of proteins similar to Fc receptors that play an important role in maintaining the homeostasis balance of the immune system (Li et al., 2014b). The *FCRL* genes are members of the immunoglobulin gene superfamily, discovered by multiple teams using different strategies, and scholars eventually designated them as a uniform nomenclature (Liu et al., 2020). In recent years, a large of studies have found that FCRLs play a vital role in immunodeficiencies, virus infections, and autoimmune diseases (Ehrhardt and Cooper, 2011; Li et al., 2014a; Davis, 2020). FCRL5 is one of the FCRLs family and contains a long, highly acidic C-terminal tail, which mediates DNA binding in a non-sequence-specific manner. FCRL5 can regulate immune response by affecting the signal transduction of immune cell receptors (Zhu et al., 2013). Genetic mutations in the *FCRL5* gene may affect the transcription and translation processes, thus altering its protein expression levels and modifying its protein function (Liu et al., 2020). Previous studies have shown that *FCRL5* gene variants were associated with several immune and inflammatory diseases such as allergic rhinitis (Gu et al., 2019), Graves’ disease (Simmonds et al., 2010), and ankylosing spondylitis (Liu et al., 2020).

In this study, we explored the associations of two *FCRL5* polymorphisms (rss6427384 and rs6692977) and *FCRL5* mRNA expression with liver fibrosis in CHB patients.

## Patients and methods

### Study subjects

A total of 114 CHB patients with liver fibrosis admitted to our hospital from January 2021 to March 2022 were selected as the study group, including 71 males and 43 females, with an average age of 52.35 ± 5.83 years. Additionally, 120 CHB patients without liver fibrosis who matched the age and gender distribution of the study group were incorporated into the control group, containing 75 males and 45 females, with an average age of 53.28 ± 6.37 years. Patients satisfying the following criteria were included: (1) the diagnosis of all subjects according to the 2019 “Guidelines for the Prevention and Treatment of Chronic Hepatitis B,” and liver fibrosis was diagnosed based on histological evaluation of liver biopsy specimens; (2) aged between 18 and 70 years; (3) clinical and laboratory data were complete. Patients with the following conditions were excluded: (1) severe kidney, cardiovascular, and cerebrovascular diseases; (2) complicated with malignant tumors, endocrine diseases, or autoimmune diseases; (3) had other types of liver diseases, such as alcoholic liver disease or hepatitis C; (4) had other viral infections, such as human immunodeficiency virus, human papillomavirus or influenza virus infections. All procedures for this study were following the Declaration of Helsinki. This research was approved by the Hospital Ethics Committee (approval number: 2021263), and informed consent was obtained from all individual participants included in the study.

### Methods

#### Collection of blood samples and clinical data

Two tubes of 5 ml fasting venous blood samples were taken from all the subjects. Refer to medical records and test sheets of them, and record their gender, age, body mass index (BMI), alanine transaminase (ALT) value, aspartate aminotransferase (AST) value, and platelet value. In addition, the aspartate aminotransferase-to-platelet ratio index (APRI) and fibrosis index based on 4 factors (FIB-4) were calculated with the following formulas:


APRI=ASTvalueIU/L/upper limit of normalASTvalueIU=L/platelet count109/L×100.

$$\begin{array}{c} \mathrm{FIB}-4=\left(\mathrm{AST}\;\mathrm{value}\;\left(\mathrm{IU}/L\right)\times \mathrm{age}\;\left(\mathrm{years}\right)\right)\\ /\left(\mathrm{platelet count}\;\left(10^{9}/L\right)\times \sqrt{\mathrm{ALT}\left(U/L\right)}\right). \end{array}$$

#### DNA sample extraction and genotyping

By reviewing the previous literature and the HapMap databases for Chinese Han population in Beijing (HapMap Data Rel 28 PhaseII + III, on NCBI B36 assembly, dpSNP b126), we selected two SNPs (rs6427384 and rs6692977) in *FCRL5* gene. The two SNPs were genotyped through the polymerase chain reaction-restriction fragment length polymorphism (PCR-RFLP) method. Genomic DNA was extracted from peripheral venous blood of all subjects using a QIAGEN kit (QIAGEN, Hilden, Germany) based on the manufacturer’s instructions, and stored at −20°C before the genotyping detection. All SNPs were genotyped using the improved Multiplex Ligase Detection Reaction (iMLDR) Assay technology (Shanghai Genesky Bio-Tech Co, Ltd.; www.Geneskies.com). The primers are as follows: rs6427384, forward: 5′-GAGCATTCAGGGAACTACTA-3′; reverse: 5′-TGGAGGAGGATATTAGGTTG-3′; rs6692977, forward: 5′-CGGTCTCACTGGGCTAAA-3′; reverse: 5′-TGACTTTGCTGGCTTTGG-3′.

#### Measurement of *FCRL5* mRNA expression

Peripheral blood mononuclear cells (PBMCs) from peripheral blood of all subjects were isolated by Ficoll–Hypaque density gradient centrifugation method. Total cellular RNA was extracted using miRNeasy Mini Kit (Qiagen, Germany). The quantification and concentration of RNA were determined using NanoDrop™ 2000 Spectrophotometer (Thermo Fisher Scientific, Wilmington, DE, United States), and RNA was reverse transcribed into complementary DNA using a PrimeScript™ RT reagent kit (Takara Bio Inc., Japan). The *FCRL5* mRNA expression levels were detected in the quantitative Real-Time PCR System (Applied Biosystems, Foster City, CA, United States) using the SYBR Green kit (Takara Bio Inc., Japan). The relative expression levels of *FCRL5* mRNA were normalized to the internal control U6 and were calculated by 2^−ΔΔCT^. The primer sequences of FCRL5 and U6 are as follows: FCRL5, forward: 5′-GTGCAAGTGTAGATGCCGACAA-3′; reverse: 5′- GTGCAAGTGTAGATGCCGACAA - 3′; U6, forward: 5′-CTCGCTTCGGCAGCACA-3′; reverse: 5′- AACGCTTCACGAATTTGCGT - 3′.

#### Statistical analysis

Statistical analyses were conducted using SPSS version 23.0 (SPSS Inc., Chicago, IL, United States). Continuous data with normally distribution were displayed as mean ± standard deviation (SD), and the comparison of normal distributed data between two groups was using the t-test. Continuous data with abnormal distribution were displayed as median (interquartile range, IQR), and data between two groups were compared using the Mann–Whitney *U* test. Categorical data were presented as absolute numbers and percentages. The distributions of genotype, genetic models, and allele frequencies in different groups were compared using the Chi-square test, and odds ratios (ORs) with 95% confidence intervals (CIs) were calculated. Hardy–Weinberg equilibrium (HWE) was assessed by the Chi-square test. The correlation between the two continuous variables was calculated by Pearson or Spearman correlation test. The receiver-operating characteristic (ROC) curve and the area under the curve (AUC) were applied to evaluate the predictive value of the variables for liver fibrosis, *p*-value <0.05 was considered as statistically significant.

## Results

### Comparison of demographic and clinical characteristics

As shown in Table 1, there were no statistically significant differences in gender, age, and BMI between fibrosis and non-fibrosis patients (all *p* > 0.05). ALT, AST, APRI, and FIB-4 in patients with fibrosis were significantly higher than those in non-fibrosis patients (all *p* < 0.05).

**Table 1:** Comparison of demographic and clinical characteristics between fibrosis and non-fibrosis group.

Characteristics	Fibrosis group (*n* = 114)	Non-fibrosis group (*n* = 120)	*χ*^2^/*t*/*Z*	*p*
Gender (male/female)	71/43	75/45	0.001	0.972
Age (year)	52.15 ± 6.21	53.27 ± 5.85	1.420	0.157
BMI (kg/m^2^)	24.24 ± 3.06	23.83 ± 2.73	1.084	0.280
ALT (U/L)	51.10 ± 12.91	35.44 ± 7.97	11.097	<0.001
AST (U/L)	49.18 ± 12.59	29.66 ± 9.77	13.202	<0.001
APRI	0.61 (0.49, 0.85)	0.31 (0.22, 0.42)	9.299	<0.001
FIB-4	2.33 (1.66, 3.08)	1.34 (1.02, 1.96)	6.938	<0.001

### Comparison of genotype distributions and inheritance models

The distributions of the two polymorphisms rs6427384 and rs6692977 were all confirmed to Hardy–Weinberg equilibrium (HWE) in the two groups. The distribution of genotype of the two SNPs in fibrosis and non-fibrosis patients is displayed in Table 2. There was a statistically significant difference between fibrosis and non-fibrosis groups in the genotype distribution of *FCRL5* rs6692977 (*p* = 0.020). While no statistically significant difference in the genotype distribution was found between the two groups at rs6427384 (*p* = 0.215).

**Table 2:** Comparison of genotype distribution and genetic models of *FCRL5* gene SNPs between fibrosis and non-fibrosis group.

Genotype	Fibrosis group (*n* = 114)	Non-fibrosis group (*n* = 120)	*χ* ^2^	*p*	OR (95% CI)	*p*
rs6427384						
TT	69 (60.53%)	85 (70.83%)	3.073	0.215	1.00 (reference)	
TC	35 (30.70%)	29 (24.17%)			1.487 (0.828–2.671)	0.183
CC	10 (8.77%)	6 (5.00%)			2.053 (0.711–5.931)	0.177
CC + TC vs. TT					1.584 (0.919–2.729)	0.097
CC vs. TC + TT					1.667 (0.584–4.753)	0.335
rs6692977						
CC	52 (45.61%)	76 (63.33%)	7.805	0.020	1.00 (reference)	
CT	46 (40.35%)	35 (29.17%)			1.921 (1.093–3.375)	0.023
TT	16 (14.04%)	9 (7.50%)			2.598 (1.067–6.324)	0.031
TT + CT vs. CC					2.059 (1.221–3.475)	0.006
TT vs. CT + CC					2.014 (0.852–4.762)	0.106

Dominant, recessive, and co-dominant genetic models in fibrosis patients were compared with that in non-fibrosis patients. The results displayed that when compared with CC genotype, the genotype CT or TT at rs6692977 was significantly associated with a increased risk of liver fibrosis in CHB patients (CT vs. CC: OR = 1.921, 95% CI = 1.093–3.375, *p* = 0.023; TT vs. CC: OR = 2.598, 95% CI = 1.067–6.324, *p* = 0.031). In addition, the dominant model was also statistically significant. That is, CHB patients who carried CT or TT genotype had a higher risk of liver fibrosis than those carried who CC genotype (OR = 2.059, 95% CI = 1.221–3.475, *p* = 0.006). Yet, for rs6427384, the dominant, recessive, and co-dominant genetic models were not statistically significant between fibrosis and non-fibrosis groups (all P>0.05).

### Comparison of allele frequencies

We further compared the allele frequencies of *FCRL5* gene SNPs between the two groups. There was a statistically significant difference in the frequency of allele C and T at rs6692977 between fibrosis and non-fibrosis groups. The occurrence of allele T significantly increased the risk of liver fibrosis in CHB patients (OR = 5.423, 95% CI = 3.209–9.165, *p* < 0.001), as demonstrated in Table 3. However, no statistically significant difference in the allele frequency at rs6427384 was observed between fibrosis and non-fibrosis patients (*p* > 0.05).

**Table 3:** Comparison of allele frequencies of *FCRL5* gene SNPs between fibrosis and non-fibrosis group.

Allele	Fibrosis group (*n* = 114)	Non-fibrosis group (*n* = 120)	*χ* ^2^	*p*	OR (95% CI)
rs6427384					
T	173 (75.88%)	199 (82.92%)	3.554	0.059	1.00 (reference)
C	55 (24.12%)	41 (17.08%)			1.543 (0.981–2.427)
rs6692977					
C	150 (65.79%)	219 (91.25%)	13.252	<0.001	1.00 (reference)
T	78 (34.21%)	21 (8.75%)			5.423 (3.209–9.165)

### Comparison of *FCRL5* mRNA expression levels

**Figure 1:**
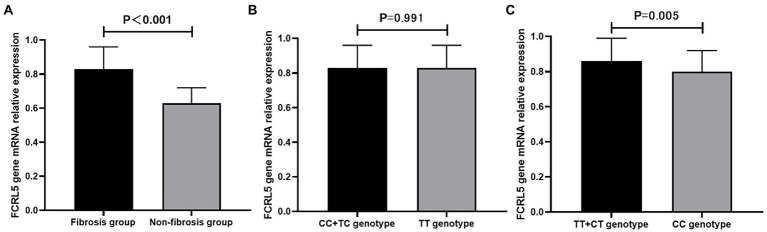
Comparison of *FCRL5* mRNA expression levels. **(A)** Comparison between fibrosis and non-fibrosis group. **(B)** Comparison between different rs6427384 genotypes. **(C)** Comparison between different rs6692977 genotypes. FCRL5, Fc receptor-like 5.

To confirm the results that *FCRL5* gene polymorphisms were associated with liver fibrosis, we measured the *FCRL5* mRNA expression levels in all subjects using the qRT-PCR assay. As shown in Figure 1A, the mRNA relative expression levels of *FCRL5* in patients with liver fibrosis were 0.83 ± 0.13, which were significantly higher than those in the non-fibrosis group (0.63 ± 0.09), with a statistically significant difference (*t* = 13.456, *p* < 0.001).

Additionally, to verify the influence of *FCRL5* SNPs on gene mRNA expression, we compared the *FCRL5* mRNA expression levels between fibrosis patients with different genotypes. We found that the *FCRL5* mRNA expression levels were not statistically discrepant between the different genotypes of rs6427384 (*t* = 0.012, *p* = 0.991), as demonstrated in Figure 1B. But fibrosis patients who carried TT or CT genotype of rs6692977 had significantly higher *FCRL5* mRNA expression levels than those who carried CC genotype (*t* = 2.859, *p* = 0.005), as shown in Figure 1C.

### Demographic and clinical characteristics and *FCRL5* gene variants

We compared the demographic and clinical characteristics of the fibrosis patients with different *FCRL5* gene genotypes. For rs6427384 locus, there were no significant differences in gender, age, BMI, ALT, AST, APRI, and FIB-4 between fibrosis patients who carried the C allele (CC or TC genotype) and only T allele (TT genotype) (all *p* > 0.05), as shown in Table 4. For rs6692977 locus, the ALT value of fibrosis patients with T allele (TT or CT genotype) was significantly higher than those with CC genotype (*p* < 0.05), as displayed in Table 5.

**Table 4:** Associations of rs6427384 genotypes with demographic and clinical characteristics.

Characteristics	CC + TC (*n* = 45)	TT (*n* = 69)	*χ*^2^/*t*/*Z*	*p*
Sex (male/female)	31/14	40/29	1.382	0.240
Age (year)	52.43 ± 6.01	51.97 ± 6.37	0.385	0.701
BMI (kg/m^2^)	24.59 ± 2.78	24.01 ± 3.23	0.988	0.325
ALT (U/L)	50.47 ± 13.66	51.51 ± 12.48	0.419	0.676
AST (U/L)	50.89 ± 12.47	48.06 ± 12.64	1.175	0.242
APRI	0.60 (0.48, 0.82)	0.61 (0.50, 0.87)	0.638	0.524
FIB-4	2.27 (1.69, 3.04)	2.37 (1.66, 3.35)	0.330	0.741

**Table 5:** Comparison of rs6692977 genotypes with demographic and clinical characteristics.

Characteristics	TT + CT (*n* = 62)	CC (*n* = 52)	*χ*^2^/*t*/*Z*	*p*
Sex (male/female)	39/23	32/20	0.022	0.881
Age (year)	51.82 ± 6.14	52.55 ± 6.34	0.618	0.538
BMI (kg/m^2^)	24.43 ± 3.21	24.08 ± 2.94	0.602	0.548
ALT (U/L)	53.81 ± 13.20	47.87 ± 11.88	2.504	0.014
AST (U/L)	49.85 ± 11.87	48.37 ± 13.47	0.627	0.532
APRI	0.66 (0.51, 0.85)	0.59 (0.43, 0.86)	0.999	0.318
FIB-4	1.83 (2.31, 3.01)	2.47 (1.53, 3.14)	0.188	0.851

### Demographic and clinical characteristics and *FCRL5* mRNA expression levels

**Figure 2:**
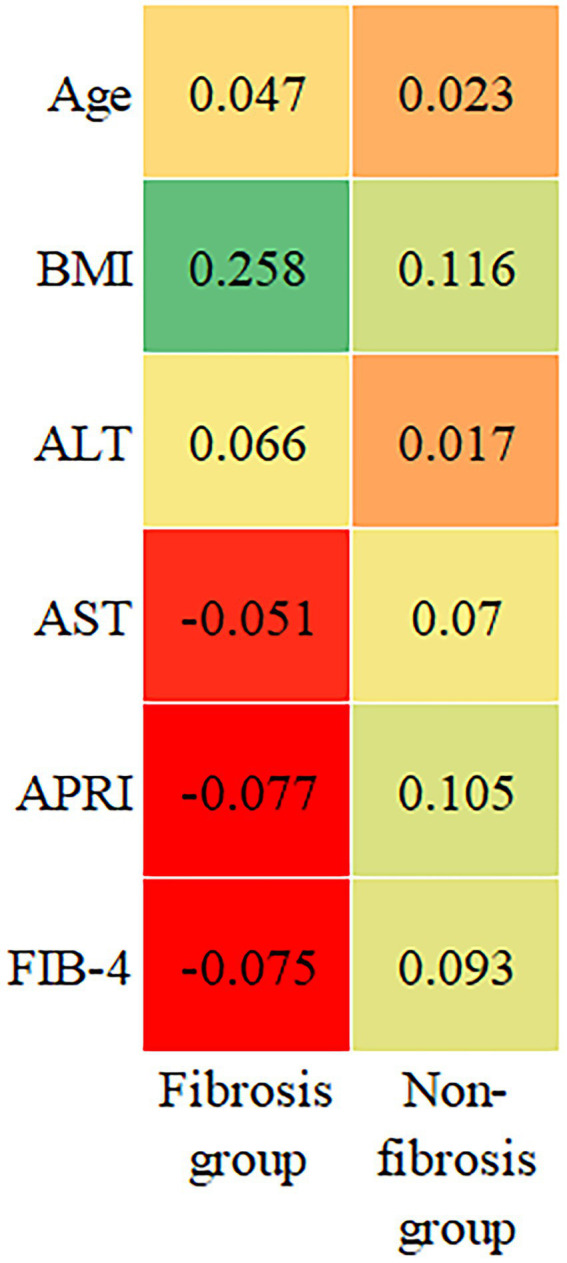
Associations of demographic and clinical characteristics with *FCRL5* mRNA expression levels. FCRL5, Fc receptor-like 5.

We analyzed the associations of *FCRL5* mRNA expression levels and demographic and clinical parameters in fibrosis and non-fibrosis patients, respectively (Figure 2). In the fibrosis group, BMI was positively correlated with *FCRL5* mRNA expression levels (*r* = 0.258, *p* = 0.006), and age, ALT, AST, APRI, and FIB-4 had no significant correlation with FCRL5 gene mRNA expression levels (all *p* > 0.05). In the non-fibrosis group, no significant correlations were observed between the demographic and clinical characteristics and *FCRL5* mRNA expression levels (all *p* > 0.05).

### Predictive effectiveness by ROC curve analysis

**Figure 3:**
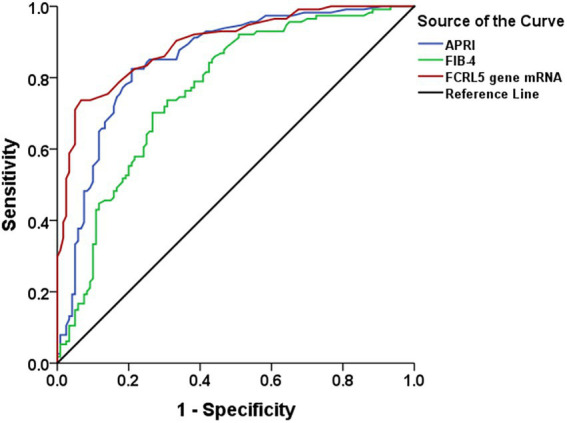
ROC curve of the *FCRL5* mRNA expression, APRI and FIB-4 index for predicting liver fibrosis in CHB patients. FCRL5, Fc receptor-like 5; ROC, receiver operator characteristic.

To evaluate the predictive value of *FCRL5* mRNA expression levels, APRI and FIB-4 index for liver fibrosis in CHB patients, ROC Curve analysis was conducted. Whether liver fibrosis occurred was considered as the state variable (non-fibrosis = 0, fibrosis = 1), and *FCRL5* mRNA expression, APRI and FIB-4 index were regarded as test variables, respectively. The mRNA expression levels of *FCRL5*, APRI and FIB-4 index showed significantly predictive value in liver fibrosis with cut-off values of 0.75 (AUC = 0.896, 95% CI = 0.856–0.935, sensitivity = 73.7%, and specificity = 93.3%), 0.45 (AUC = 0.852, 95% CI = 0.802–0.902, sensitivity = 82.5%, and specificity = 79.2%) and 1.84 (AUC = 0.765, 95% CI = 0.703–0.826, sensitivity = 70.2%, and specificity = 73.3%), respectively. The maximum of the Youden index for FCRL5 mRNA expression, APRI and FIB-4 in the prediction of liver fibrosis were 0.670, 0.617 and 0.435, respectively, as displayed in Figure 3.

## Discussion

The outcome of HBV infection is influenced by the host immune system, and immunologic injury is an important factor influencing the occurrence and development of liver fibrosis in CHB patients (Bandopadhyay and Bharadwaj, 2020; Wu et al., 2020). FCRLs are a class of proteins similar to Fc receptors, which play an important role in maintaining the homeostasis of the immune system (Tolnay, 2022). *FCRL* gene belongs to the immunoglobulin gene superfamily, of which *FCRL5* is an important member and has been reported to play a crucial part in the regulatory mechanism of immune cells (Liu et al., 2020). A previous study demonstrated that FCRL5 exerts binary and compartment-specific influence on innate-like B-cell receptor signaling, and implied a specialized counterregulatory role for FCRL5 at the intersection of innate and adaptive immunity (Zhu et al., 2013). Ogega and Skinner (2022) observed that B cell overexpression of FCRL5 was associated with low antibody titers in HCV infection, and limited upregulation of FCEL5 could potentially generate higher titers of protective antibodies against HCV or other pathogens. In addition, in recent years, many studies have found that FCRL5 was associated with several diseases. Zhang et al. (2022) revealed that FCRL5 was an independent risk factor for colorectal cancer by bioinformatics analysis. Guzeldemir-Akcakanat et al. (2016) found that the expression of FCRL5 was up-regulated in Generalized Aggressive Periodontitis. In addition, it has been suggested that FCRL5 may promote malignant cell growth in hairy cell leukemia and other tumors expressing FCRL5 (Dement-Brown et al., 2012). Additionally, Cell proliferation and downstream isotype expression were enhanced under FCRL5 stimulation, reflecting the physiological role of FCRL5 in antigen-initiated B cell expansion and development (Dement-Brown et al., 2012). The expression of a gene may be regulated by its genetic polymorphisms. Gene mutations may affect the binding of the region where the site is located with transcription factors, thus affecting the transcription process of mRNA. Previous studies have reported that SNPs of multiple genes are related to the occurrence, development, and prognosis of HBV-related fibrosis (Guo et al., 2015; Han et al., 2020; Su et al., 2022). The purpose of this study was to investigate the relationship between *FCRL5* gene variations and liver fibrosis in CHB patients and its effect on gene mRNA expression levels.

Previous studies indicated significant relationships of *FCRL5* gene variants with plenty of inflammatory or immunological disorders. Gu et al. (2019) showed that *FCRL5* gene polymorphism is closely related to the occurrence of allergic rhinitis combined with asthma in the Chinese population. Compared with healthy control group, the proportion of CT genotype and T allele frequency of rs6692977 in asthmatic patients with allergic rhinitis was significantly increased. Simmonds et al. (2010) found that rs6692977 mutation was associated with the occurrence of hyperthyroidism, with a higher frequency of the T allele in the case group compared to the control population. Liu et al. (2020) showed that rs6427384 polymorphisms of the *FCRL5* gene were associated with the incidence of ankylosing spondylitis in the Chinese Han population, and CC genotype and C allele of rs6427384 locus were significantly reduced in the case group when compared with the healthy control population. The results of our study revealed the associations between rs6692977 but not rs6427384 mutations with liver fibrosis in CHB patients, which suggest that to a certain extent, hepatitis B-related fibrosis have similarity and differences in genetic background with other disorders with immune dysfunction.

In addition, this study observed that the mRNA expression levels of *FCRL5* in CHB patients with liver fibrosis were significantly higher than those in non-fibrosis patients. The *FCRL5* mRNA expression levels in patients with TT or CT genotype at rs6692977 were significantly higher than those in patients with CC genotype. This result reveals that the *FCRL5* mRNA expression levels are influenced by rs6692977 polymorphisms. Then we found that the ALT value of fibrosis patients with TT or CT genotypes was significantly higher than those with CC genotypes. Hence, *FCRL5* gene variants may be associated with liver function. The study of Sun et al. (2021) showed that the expression of FCRL5 protein was significantly up-regulated in patients with liver cancer, and it could promote the proliferation, invasion and migration of liver cancer cells. The mechanism may be related to the high expression of matrix metalloproteinase-9 and vascular endothelial growth factor induced by FCRL5 in liver cancer cells. Liver fibrosis is a developmental and evolutionary stage of liver cancer. Therefore, we speculated that rs6692977 TT or CT variants of the *FCRL5* gene could up-regulate gene expression levels, and thus participate in the pathogenesis of liver function injury and liver fibrosis.

Furthermore, we observed that in the fibrosis group, BMI was positively correlated with *FCRL5* mRNA expression levels. A study by Seto et al. (2016) revealed that an increased BMI hindered fibrosis regression during therapy in CHB. As we mentioned above, changes in FCRL5 expression levels are associated with immune dysregulation, which has been shown to lead to increased BMI. Hence, in the treatment of liver fibrosis, FCRL5 levels may also need to be controlled to achieve better outcomes. In recent years, APRI and FIB-4 have been widely used to diagnose liver fibrosis in CHB patients. The results of our study showed that the sensitivity and specificity of APRI for the prediction of liver fibrosis were 82.5% and 79.2%, respectively, and that of FIB-4 for the prediction of liver fibrosis were 70.2% and 73.3%, respectively. The ROC curve results of *FCRL5* mRNA expression levels for the predictive value of liver fibrosis showed that AUC was 0.896, with sensitivity and specificity of 73.7% and 93.3%. Compared with APRI and FIB-4, the specificity of *FCRL5* mRNA expression levels in predicting fibrosis was predominant, while the sensitivity was ordinary. Liver biopsy has been considered as the gold standard for the diagnosis of liver fibrosis. However, a series of defects in liver biopsy, such as large sample error, high bleeding risk, and heavy medical burden, have limited its wide application. In addition, repeated histopathology is impractical because of its inescapable invasiveness. Imaging techniques, including transient electrograph (TE), shear wave elastography (SWE), acoustic radiation pulse imaging (ARFI), and magnetic resonance elastography (MRE) have good diagnostic efficacy for liver fibrosis (Pfeifer et al., 2015; Ragazzo et al., 2017; Xiao et al., 2017; Herrmann et al., 2018). However, they are expensive, and the diagnostic accuracy is easily affected by a series of factors such as obesity, ascites, acute inflammation, liver congestion, portal hypertension and so on, which reduces the credibility of the diagnostic results (Tsochatzis et al., 2011; Yin et al., 2011). Compared to liver biopsy and imaging methods, laboratory tests are non-invasive, cheap and have better repeatability. Although the efficacy of *FCRL5* mRNA expression levels in predicting liver fibrosis was mediocre, it can provide a new perspective for predicting liver fibrosis by non-invasive method.

However, there are still some limitations in the current study. Firstly, the sample size of this study was small, so selection bias was inevitable. Secondly, the levels of laboratory indicators are closely related to the medication status of patients, which has not been analyzed. In the future, if the conditions permit, we will increase the sample size and conduct more rigorous and detailed analyses. Thirdly, we did not conduct functional experiments to investigate the exact mechanisms of FCRL5 gene variations in the occurrence and development of liver fibrosis. We will explore this aspect further in the future when conditions are available.

In conclusion, *FCRL5* gene rs6692977 polymorphisms and mRNA expression levels are associated with liver fibrosis in CHB patients.

## Data availability statement

The data that support the findings of this study are available from the corresponding author upon reasonable request.

## Ethics statement

The studies involving human participants were reviewed and approved by the Affiliated Suzhou Hospital of Nanjing Medical University. The patients/participants provided their written informed consent to participate in this study.

## Author contributions

YW is the guarantor of this work. JG, HW, JS, LL, and WS: specimen and data collection. JY: statistical analysis and manuscript writing. JY and JG: article revising. All authors contributed to the article and approved the submitted version.

## Funding

This study was funded by grants from the Science and Technology Development Project of Nanjing Medical University (NMUB2020259).

## Conflict of interest

The authors declare that the research was conducted in the absence of any commercial or financial relationships that could be construed as a potential conflict of interest.

## Publisher’s note

All claims expressed in this article are solely those of the authors and do not necessarily represent those of their affiliated organizations, or those of the publisher, the editors and the reviewers. Any product that may be evaluated in this article, or claim that may be made by its manufacturer, is not guaranteed or endorsed by the publisher.
